# Splenic rupture after colonoscopy: Report of a case and review of literature

**DOI:** 10.1186/1749-7922-3-8

**Published:** 2008-02-09

**Authors:** Alessandro Cappellani, Maria Di Vita, Antonio Zanghì, Andrea Cavallaro, Giovanni Alfano, Gaetano Piccolo, Emanuele Lo Menzo

**Affiliations:** 1 University of Catania Medical School, Policlinico, Department of Surgery, Catania, Italy; 2 University of Catania Medical School, Policlinico, Fellowship in Surgical Physio-Pathology, Catania, Italy; 3 University of Miami, Miller School of Medicine, Department of Surgery, Miami, Florida, USA

## Abstract

Splenic rupture is a rare complication of colonoscopy. For this reason the diagnosis could be delayed and the outcome dismal. Fifty-four cases of splenic rupture after colonoscopy have been described in the literature. The majority of the cases required emergent or delayed splenectomy, 13 of these cases were treated conservatively. The main feature that stands out from the review of the literature is the "surprise" of this unexpected complication. This factor explains the elevated mortality (2 out of 54 cases), likely due to the delay in diagnosis. The case here described is probably among the most complex published in the literature; in fact the presence of dense intra-abdominal adhesions not only contributed to the complication itself, but also explain the confinement of the hemoperitoneum to the left supra-mesocolic space and the delayed presentation (13 days from the time of the trauma).

## Background

Colonoscopy is a very popular diagnostic and therapeutic procedure, and it is usually very well tolerated by the patient. Besides the complications due to the bowel preparation (abdominal pain, volume overload [1]) and the peri-procedure sedation (respiratory depression, allergic reactions), the most common complications of colonoscopy are perforation (0.34%–2.14%) and hemorrhage (1.8–2.5%). Other less frequent complications have been described, such us: pneumothorax, pneumoperitoneum, volvulus, hernia incarceration, and retroperitoneal abscess [2].

Finally, acute appendicitis and splenic rupture are extremely rare complications. Only nine cases of acute appendicitis after colonoscopy have been reported. The pathophysiologic mechanism seems to be due to the luminal occlusion by a fecalith during the endoscopic maneuvers [3].

The first case of splenic rupture after colonoscopy was published in 1975 by Wherry and Zehner [4]. At the time of this report, a total of 54 cases of splenic rupture after colonoscopy have been described in 49 reports, 43 of which in English language [1-49]. In the majority of the cases the clinical manifestation was with diffuse peritonitis and hypovolemic shock within 24 hours of the endoscopic procedure.

In rare cases the diagnosis is made after 48–72 hours. Our case of a two-stage splenic rupture after 13 days seems exceedingly rare

## Methods

### Case Report

The patient is a 50-year old woman admitted at our Department of General and Breast Surgery of the University of Catania.

The patient had an extensive past surgical history that included a cholecystectomy in 1984, a right upper quadrantectomy with lymphoadenectomy in 1997, a left quadrantectomy in 1998, and a radical total abdominal hysterectomy in 2003 for large fibromas.

Eleven days prior to the admission to our unit, the patient underwent colonoscopy for a history of rectorrhagia. The exam was conducted under light sedation and without difficulties. The exam was unremarkable to the cecum except for the presence of several diminutive polyps (3 mm) in the rectum, which were biopsied for histological evaluation. At the end of the procedure the patient developed left sided chest pain and a syncopal episode that she did not report to her family or to her physician.

The following day the patient had a mild fever, but she was otherwise stable.

On post-procedure day 4 the patient went to her family physician, who obtained a chest and abdominal radiographic series and some routine blood work. The chest roentegram revealed an obliteration of the left costophrenic angle, whereas the abdominal radiograph was within normal limits. The laboratory analysis revealed a normal hemoglobin level (14 g/dl) and a mild leukocytosis (17.4). The patient denied any spontaneous or induced abdominal pain, the bowel function was within normal limits, but she had a persistent modest temperature elevation (38 degrees).

The patient was then treated with antibiotics and analgesics. A second chest roentegram appeared unchanged.

Because of her persistent and reproducible left sided chest pain and fever and her history a previous mastectomy for cancer, she was referred to us because of a suspected pleuro-parenchymal lesion.

**Figure 1:**
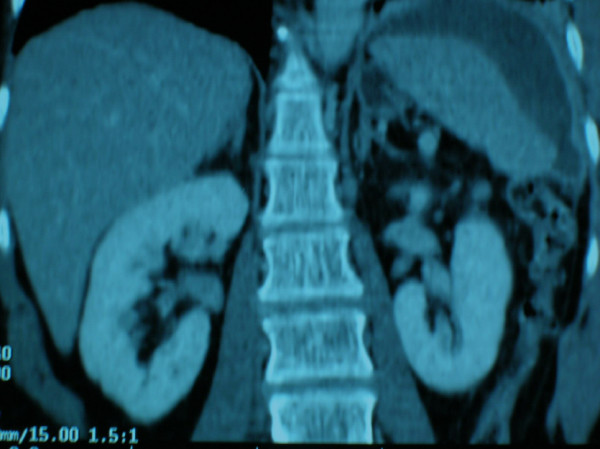
**CT scan**. The computed tomography of the chest and abdomen showed some atelectasis at the left base with hemi-diaphragmatic elevation and hypodense areas, convex in shape, within the spleen indicative of subcapsular hematoma.

After confirming her modest leukocytosis and normal hemoglobin level, she underwent a computed tomography of the chest and abdomen, which showed some atelectasis at the left lung base with hemi-diaphragmatic elevation and hypodense convex areas within the spleen, indicative of subcapsular hematoma (Figure 1). In light of her hemodynamic stability and her grade 2 splenic rupture, as per the Organ Injury Scaling Committee of the American Association for the Surgery of Trauma (AAST) [50], a non operative approach was elected. The patient was then treated with bed rest, close clinical and laboratory monitoring. On post-procedure day 13 the patient reported worsening of her previous symptomatology with signs of hemodynamic instability (Blood Pressure 80/50 mmHg, Heart Rate 125 beats/min, Respiratory Rate 28/min). The laboratory analysis revealed a decrease in her hemoglobin from 13.8 g/dl to 9 g/dl and a significant drop in her hematocrit. The ultrasonographic exam showed an intra and perisplenic area of dyshomogenity. After appropriate resuscitation with fluid and blood transfusion the patient was emergently taken to the operating room for exploration. After a tedious adhesiolysis, a localized and substantial hemoperitoneum was found in the left upper quadrant. The splenic artery was then ligated at the superior margin of the pancreas and the spleen was removed. After a thorough abdominal wash out, a closed suction drain was left in the splenic bed. The patient did not require any further transfusions after surgery and was discharged on post-operative day 6.

## Discussion

After performing a Medline search using the keyword colonoscopy, splenic rupture, splenic injury, splenic trauma, we found 49 citations, 43 of which in English language, for a total of 54 cases of splenic rupture after colonoscopy. Overall there is a female sex preponderance (sex ratio of 3.8/1) and an average age of 62 years (range 29–85). In 13 cases the patient has a previous history of intra-abdominal operations. Twenty-one of the colonoscopies were simply diagnostic, one was done in conjunction with an upper endoscopy, 3 included biopsies and 15 had concurrent polypectomies. Only three cases were described as modestly difficult.

Only one patient was on oral anticoagulation therapy with Warfarin.

The onset of symptoms was usually immediate (within 24 hours from the exam), but in some cases was delayed by several days [2,4,7,24,26,27,33,36-38,46].

Although the diagnosis of the first reported case was made by angiography, the Computed Tomography (CT scan) is the main test utilized now days. In previous series (before 1991) the most common diagnostic modality was exploratory laparotomy (10 cases), followed by ultrasonography and CT scan (2 cases), diagnostic peritoneal lavage (1 case) and post-mortem (1 case). The treatment was by emergent laparotomy in the majority of the cases, whereas 12 cases where treated non-operatively. In one case the treatment was by percutaneous embolization of the splenic artery. One reported case of a patient with a history of Crohn's disease had a combined splenic and liver laceration found on exploratory laparotomy. One patient died in spite of emergent splenectomy.

The reason for splenic rupture after a colonoscopic examination seems to be associated with the alteration of the peritoneal attachments that support the spleen in the left upper quadrant (ligaments gastrolienal, pancreaticolienal, phrenolienal).

Any kind of traction on these ligaments could determine a capsular rupture, which then disrupts a portion of the parenchyma densely adherent to it.

The rupture can be immediate because of damage to the hilum or entire organ disintegration, or delayed. In the first case the clinical presentation is with hypovolemic shock. In the latter the timing of manifestation of the shock varies and it could be gradual and delayed by few hours from the trauma depending upon degree of the parenchymal and capsular lesions. The mechanism is related to the formation of a subcapsular or intra-parenchymal hematoma, that then, instead of organizing itself into a pseudocyst, increases in size until determines the rupture of the capsule (delayed splenic rupture).

Although the exact reasons of development of a subcapsular or intra-parenchymal hematoma after a colonoscopy are not entirely clear, three mechanisms have been postulated:

The first one is related to the sudden trauma when the endoscope traverses the splenic flexure [2].

The second one is related to the avulsion of the splenic capsule caused by the excessive traction on the spleno-colic ligament during the endoscopic examination [1].

The third theory implies the traction on the adhesions between the spleen and the colon determined by previous surgical interventions or inflammatory processes [2].

In any case there are several risk factors that could predict the rupture of the spleen after colonoscopy: coagulopathies, infectious or hematological splenomegalies, specific pharmacological treatments (such as Hematopoyetic Growth Factors), intestinal or pancreatic inflammatory processes and previous intra-abdominal operations [7-9,11-13].

Other authors blame this complication to the endoscopic manoeuvres utilized to navigate through the splenic flexure (in particular hooking and reduction) or to therapeutic interventions such as polypectomies and biopsies [10,15].

In our case the colonoscopic exam was easily conducted and there was no therapeutic intervention except for a biopsy of small rectal polyps.

Differently from the cases published in the literature, in which the clinical manifestation appeared between 2 hours and 10 days [13,29], our patient presented with vague and non specific chest pain soon after the endoscopic exam, but the clinical picture related to the splenic rupture was delayed by 13 days.

In most of the cases the patients report left upper quadrant abdominal pain which can be referred to the left shoulder (Kehr sign). The latter sign is not specific and could also be present after uncomplicated colonoscopies. Only one of the reported cases presented with complete lack of pain [10]. In more typical cases, the physical finding can vary from tenderness localized at the epigastrium and left upper quadrant with reduction or absence of gastric tympanism, to diffuse peritoneal signs. Other common signs are those related to the hemodynamic alterations and vary from pallor, hypotension, tachycardia, dyspnea, to the more dramatic signs of shock.

In other cases the clinical presentation is more subtle. This usually occurs in cases of delayed rupture or in the presence of dense adhesions. The adhesions, in fact, not only can limit the extent of the hematoma, but can also prevent the onset of signs of peritoneal irritation from the hemoperitoneum.

In our case the dense adhesions, result of the previous extensive surgical history, determined the unusual and delayed clinical presentation. We can speculate, in fact, that following the trauma or stretching of the splenic ligaments (physiologic or post-operative), the dense adhesions determined a sort of hemostasis and delayed the splenic rupture with signs of hemodynamic instability only13 days after the event.

The atypical chest pain reported by the patient with the associated fever, leukocytosis and left pleural effusion was initially interpreted as a non specific pleuro-parenchymal lesion due to her previous history of bilateral breast cancer. Since the most common complication after colonoscopy are related to perforation and hemorrhage, the absence of intra-abdominal findings with negative abdominal plain x-rays, led to the exclusion of a complication related to the colonoscopy. It is then possible that a number of self-limited splenic ruptures goes undiagnosed because of lack of important symptomatology. On the other hand it is possible that other cases with an even longer lag of time between the endoscopic procedure and the splenic rupture were interpreted as a consequence of a more recent traumatic event and as such not published in the literature. It is then important to rule out a splenic rupture by U/S or CT scan in every case of abdominal or thoracic pain associated with anemia and lack of rectorrhagia [1,2,13,24].

Since both the plain abdominal x-ray and the U/S give indirect signs of splenic rupture, the CT scan is the diagnostic exam of choice, especially for those patients candidate for non-operative treatment. In the literature 9 patients underwent successful conservative treatment with transfusions and close monitoring [12,16,19,21,29,33,46,48] and only one required splenic artery embolization [32].

## Conclusion

There are only 54 cases of splenic rupture following colonoscopy published in the literature. Some authors postulate that there are other less severe cases that are not published. The rarity of this complication and the potential delay in its clinical presentation (like in the case here reported), could lead to dismal results. The presence of abdominal or thoracic pain associated with anemia and/or hypotension after a colonoscopic exam, should follow a strict diagnostic protocol in order to rule out a splenic rupture. In fact although rare this is the most insidious complication of colonoscopy. For this reason the consensus conference of the Italian Society of Surgery on "Safety in Surgery" describes the splenic rupture as a rare but severe complication and ranks it the third complication of colonoscopy after hemorrhage and colonic perforation [51].

## Abbreviations

CT – Computed tomography

U/S – Ultrasound

## Competing interests

The authors declare that they have no competing interests.

## Authors' contributions

AC, MDV, AZ, GA attended the patient, conceived the study and drafted the manuscript. AC and GP contributed to the study and participated in drafting the manuscript. ELM revised critically the manuscript for important intellectual content. All Authors read and approved the final manuscript.
